# Predicting poor functional outcomes for patients with large computed tomography perfusion core infarctions treated with endovascular thrombectomy

**DOI:** 10.1371/journal.pone.0309163

**Published:** 2024-11-18

**Authors:** Rahul R. Karamchandani, Sagar Satyanarayana, Hongmei Yang, Jeremy B. Rhoten, Dale Strong, Jonathan D. Clemente, Gary Defilipp, Nikhil M. Patel, Joe Bernard, William R. Stetler, Jonathan M. Parish, Stacey Q. Wolfe, Amy K. Guzik, Andrew W. Asimos

**Affiliations:** 1 Department of Neurology, Neurosciences Institute, Atrium Health, Charlotte, North Carolina, United States of America; 2 Information and Analytics Services, Atrium Health, Charlotte, North Carolina, United States of America; 3 Charlotte Radiology, Neurosciences Institute, Atrium Health, Charlotte, North Carolina, United States of America; 4 Department of Internal Medicine, Pulmonary and Critical Care, Neurosciences Institute, Atrium Health, Charlotte, North Carolina, United States of America; 5 Carolina Neurosurgery and Spine Associates, Neurosciences Institute, Atrium Health, Charlotte, North Carolina, United States of America; 6 Department of Neurological Surgery, Wake Forest University School of Medicine, Winston-Salem, North Carolina, United States of America; 7 Department of Neurology, Wake Forest University School of Medicine, Winston-Salem, North Carolina, United States of America; 8 Department of Emergency Medicine, Neurosciences Institute, Atrium Health, Charlotte, North Carolina, United States of America; UCSF: University of California San Francisco, UNITED STATES OF AMERICA

## Abstract

**Objective:**

Stroke patients with large core infarctions benefit from endovascular intervention, though only approximately 20% are functionally independent at 90 days. We studied prognostic factors for patients presenting with a large computed tomography perfusion (CTP) core.

**Methods:**

Retrospective analysis from a health system stroke registry, including consecutive thrombectomy patients treated within 24 hours from August 2020-December 2022 with an anterior circulation large vessel occlusion and CTP core infarct ≥50 milliliters. Logistic regression was used to determine independent predictors of 90-day modified Rankin Scale (mRS) score 4–6. The prognostic ability of previously reported scales was also assessed.

**Results:**

In 118 included patients, with mean age 64.3 ± 14.1 years, poor functional outcomes were present in 66 subjects (55.9%). The multivariable regression analysis demonstrated that higher presenting National Institutes of Health Stroke Scale (NIHSS) score (odds ratio [OR] 1.12, 95% confidence interval [CI] 1.02–1.23, p = 0.014), elevated glucose (OR 1.02, 95% CI 1.01–1.03, p = 0.002), absence of treatment with intravenous thrombolysis (OR 4.01, 95% CI 1.35–11.95, p = 0.013), and poor revascularization (OR 4.76, 95% CI 1.24–18.37, p = 0.023) were independently associated with primary outcome. The Charlotte Large artery occlusion Endovascular therapy Outcome Score (CLEOS) predicted 90-day mRS 4–6 (per 25-point increase, OR 1.22, 95% CI 1.10–1.34, p<0.001) and mRS 5–6 (per 25-point increase, OR 1.21, 95% CI 1.10–1.33, p<0.001). Nineteen of 20 (95%) patients with CLEOS ≥ 675 had 90-day mRS scores of 4–6, while 10 of 12 (83.3%) with CLEOS ≥ 725 had 90-day mRS scores of 5–6.

**Conclusion:**

We report prognostic factors that can risk stratify thrombectomy patients with large CTP core infarctions.

## Introduction

Stroke patients presenting with large core infarctions were predominantly excluded from early- and extended-window endovascular thrombectomy (EVT) randomized controlled trials (RCTs) [1–3]. However, previously published data suggest that thrombectomy may be beneficial in this population based on a meta-analysis of early-window studies [4], a systematic review [5], a secondary analysis of a prospective trial [6], a retrospective analysis of a prospectively collected stroke registry [7], and a randomized trial in Japan [8].

More recently, 3 trials have demonstrated the efficacy of EVT in patients presenting with large core infarctions [9–11]. In SELECT2 (Randomized controlled trial to optimize patient’s selection for endovascular treatment in acute ischemic stroke), patients presenting within 0–24 hours with an internal carotid artery (ICA) or proximal middle cerebral artery (MCA) occlusion and large core infarct (Alberta Stroke Program Early Computed Tomography Score [ASPECTS] 3–5 or computed tomography perfusion [CTP] core 50 milliliters [ml] or greater) had improved functional outcomes at 90 days compared to those treated with medical management [9]. Similarly, subjects presenting with an anterior circulation large vessel occlusion and ASPECTS 3–5 or CTP core 70–100 ml benefited from thrombectomy in ANGEL-ASPECT (Endovascular therapy in acute anterior circulation large vessel occlusive patients with a large infarct core) [10]. Meanwhile, TENSION (Endovascular thrombectomy for acute ischemic stroke with established infarct: multicenter, open-label, randomized trial) enrolled patients with ASPECTS 3–5 within 12 hours of onset and showed functional outcome and mortality benefits in favor of thrombectomy at 90 days [11].

While these pivotal trials are encouraging in that they demonstrate the efficacy of EVT in a larger subset of large vessel occlusion patients, only 17–30% of study subjects treated with thrombectomy were functionally independent at 90 days [9–11]. Although thrombectomy has clear efficacy in the large core population, a substantial proportion of patients are left dead or dependent despite endovascular treatment.

In this study, we examine the factors associated with poor outcomes in patients presenting with large CTP core infarctions, a subset of the population enrolled in large core randomized trials. Additional prognostic factors may influence pre- and post-thrombectomy decision-making by identifying patients at high likelihood of experiencing poor outcomes. We also study the performance of several previously reported prognostic scales in patients presenting with a large ischemic core.

## Materials and methods

### Inclusion criteria and outcomes

We queried our health system code stroke registry, which includes approximately 7000 stroke patients annually, for ICA and MCA occlusion patients presenting between August 2020 and December 2022 with CTP core infarct (cerebral blood flow [CBF] < 30% compared to contralateral hemisphere) of at least 50 ml treated with EVT within 24 hours of the time last known to be in their usual state of health. The primary outcome measure was 90-day mRS score 4–6, with higher scores indicating worse functional outcomes [12].

The study was approved by the health system Institutional Review Board (File #06-21-02E). Due to the retrospective nature of the analysis, informed consent was not required by the board. The data that support the findings in this analysis are available as a supplemental file to this manuscript. The first author (RRK) and another author (DS) had access to individual patient identifiers during data collection. The data were accessed for research purposes from 1 March 2023–1 April 2023.

### Definition of variables

Demographic data were captured from the electronic medical record. The NIHSS score was calculated by the treating clinical team. Glucose was recorded as the fingerstick blood sugar or first laboratory value. Automated software (RAPID [iSchemaView, Inc., Menlo Park, CA, USA] or Viz.ai [San Francisco, CA, USA]) was applied to the CTP performed at the presenting hospital for calculation of the ischemic core, delayed perfusion (time-to-maximum greater than 6 seconds, Tm > 6), mismatch volume (Tm > 6 minus core) and mismatch ratio (Tm > 6 divided by core). Cerebral blood volume (CBV) index (the average CBV in hypoperfused tissue divided by the average CBV in tissue with normal perfusion) and the hypoperfusion intensity ratio (HIR, time-to-maximum greater than 10 seconds divided by Tm > 6), both indicators of collateral blood flow, were recorded from automated CTP processing. Good reperfusion was defined as modified TICI (mTICI) 2b-3.

### Endovascular thrombectomy

Patients were treated with EVT based on the health system guideline (S1 Table), modeled after guidelines endorsed by the American Heart Association/American Stroke Association [13]. The health system includes both a Joint Commission certified Comprehensive Stroke Center and a Thrombectomy Capable Center. Thrombectomy decision-making was made by the treating Neurology team and Neuro-interventionalist. EVT techniques may have included aspiration, stent-retriever, intra-arterial thrombolysis, or a combination of these interventions. Anesthesia technique (conscious sedation or general) was used at the discretion of the treating Neuro-interventionalist.

### Statistical analysis

Independent variables for the entire cohort and stratified by outcome (mRS 0–3 versus mRS 4–6) are reported as mean (standard deviation, SD), median (interquartile range, IQR), or frequencies (percentages). The Chi-square test or Fisher’s exact test was used for categorical variables and continuous variables were evaluated with the T-test for normally distributed variables or the Mann-Whitney U-test for variables with a non-normal distribution.

Univariate association of each independent variable with the primary outcome was assessed using simple logistic regression analyses. Factors significant at p<0.10 in the univariate analyses, along with age and time to CTA, were included in a multivariable logistic regression to assess their independent association with the outcome. The ability of the previously reported Charlotte Large artery occlusion Endovascular therapy Outcome Score (CLEOS) [14], Houston Intra-Arterial Therapy 2 (HIAT-2) [15], Pittsburgh Response to Endovascular therapy (PRE) [16], Totaled Health Risks in Vascular Events (THRIVE) [17], and Stroke Prognostication using Age and NIHSS (SPAN-100) [18] (S2 Table), to predict 90-day mRS 4–6 and 90-day mRS 5–6 was assessed using simple logistic regression and area under the curve (AUC) analyses. The Delong test was used to compare AUC values of each scale.

## Results

**Fig 1:**
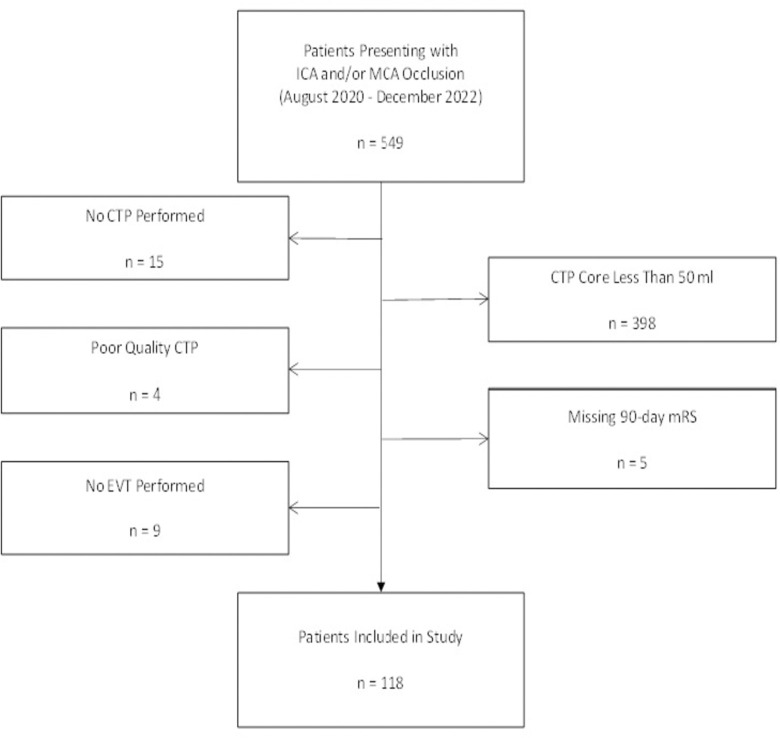
Patient flow diagram for study inclusion. ICA, internal carotid artery; MCA, middle cerebral artery; n, number; CTP, computed tomography perfusion; ml, milliliters; EVT, endovascular thrombectomy; mRS, modified Rankin Scale.

One-hundred eighteen patients met inclusion criteria (Fig 1), of whom 66 (55.9%) had an unfavorable outcome (mRS 4–6; Table 1). Forty-one of 118 (34.7%) were functionally independent (mRS 0–2) at 90 days. Approximately 61% of the total included subjects were male and the median presenting NIHSS was 19 (interquartile range [IQR] 15–23). Median CTP core size for the entire cohort was 76.5 ml (IQR 64.25–107). Twenty-two of 118 patients (18.6%) had CTP scans performed within 1 hour, while mean time to revascularization for the entire cohort was just under 6 hours (Table 1).

**Table 1:** Relationship between patient characteristics and 90-Day functional outcome.

	All patients, N = 118	mRS 0–3, N = 52 (44.1%)	mRS 4–6, N = 66 (55.9%)	p-value^
Age, years, mean ± SD	64.31 ± 14.13	62.46 ± 13.10	65.77 ± 14.83	0.2011
Sex, male, n (%)	72 (61.02)	36 (69.23)	36 (54.55)	0.1516
Race				
Black	35 (29.66)	17 (32.69)	18 (27.27)	0.7670
White	77 (65.25)	33 (63.46)	44 (66.67)	
Other/unknown	6 (5.08)	2 (3.85)	4 (6.06)	
Hypertension, n (%)	80 (67.8)	40 (76.92)	40 (60.61)	0.0920*
Hyperlipidemia, n (%)	54 (45.76)	27 (51.92)	27 (40.91)	0.3143
Diabetes mellitus, n (%)	36 (30.51)	13 (25)	23 (34.85)	0.3410
Atrial Fibrillation, n (%)	24 (20.34)	8 (15.38)	16 (24.24)	0.3388
Smoking, n (%)	46 (38.98)	21 (40.38)	25 (37.88)	0.9307
Coronary artery disease, n (%)	23 (19.49)	10 (19.23)	13 (19.7)	1.0000
Presentation to EVT center, n (%)	69 (58.47)	29 (55.77)	40 (60.61)	0.7329
Initial NIHSS, median (IQR)	19 (15–23)	18 (15–22.25)	20 (16–24)	0.0602*
Glucose (mg/dL), mean ± SD	144.79 ± 68.66	119.15 ± 34.18	164.98 ± 81.42	< 0.001*
Site of Occlusion, n (%)				0.5062
Internal Carotid Artery	39 (33.05)	15 (28.85)	24 (36.36)	
Middle Cerebral Artery	79 (66.95)	37 (71.15)	42 (63.64)	
Left hemisphere	62 (52.54)	25 (48.08)	37 (56.06)	0.4987
Right hemisphere	56 (47.46)	27 (51.92)	29 (43.94)	
CT ASPECTS, median (IQR)	10 (8–10)	9 (8–10)	10 (7.25–10)	0.9907
CBF < 30% (ml), median (IQR)	76.5 (64.25–107)	72 (59.5–99.25)	88.5 (65.25–115.75)	0.0245*
Tm > 6s (ml), median (IQR)	173 (132.5–225)	174.5 (143–216.75)	171 (131.25–236.5)	0.6883
Mismatch volume (ml), median (IQR)	91.5 (53.25–128.50)	98 (59.75–130.75)	86.5 (48.25–126.75)	0.4255
Mismatch ratio, median (IQR)	2.15 (1.60–2.80)	2.25 (1.6–3.1)	2 (1.5–2.6)	0.1100
HIR, median (IQR)	0.6 (0.6–0.7)	0.6 (0.6–0.7)	0.7 (0.6–0.8)	0.5011
CBV index, median (IQR)	0.6 (0.5–0.7)	0.7 (0.6–0.8)	0.6 (0.5–0.7)	0.0375*
Intravenous thrombolysis, n (%)	57 (48.31)	32 (61.54)	25 (37.88)	0.0179*
TLKW to CTA (min) mean±SD	231.89 ± 251.8	224.47 ± 254.72	237.62 ± 251.32	0.5201
TLKW to final revasc (min) mean±SD	357.57 ± 258.72	341.2 ± 267.18	370.23 ± 253.31	0.2621
Post-treatment mTICI 2b-3, n (%)	97 (82.2)	47 (90.38)	50 (75.76)	0.0688*
Symptomatic ICH, n (%)	5 (4.24)	0 (0)	5 (7.58)	0.0659*

^ T-test for age (normally distributed) and Mann-Whitney U test for other continuous variables; Fisher’s exact test for Race and Symptomatic ICH, and Chi-square test for other categorical variables.

*Significant p-value (p<0.10). Note: not all baseline characteristics with p<0.10 in Table 1 were significantly different between groups in the univariate analysis.

ml, milliliters; mRS, modified Rankin score; SD, standard deviation; EVT, endovascular thrombectomy; NIHSS, National Institutes of Health Stroke Scale; mg, milligrams; dL, deciliter; CT, Computed Tomography; ASPECTS, Alberta Stroke Program Early Computed Tomography Score; CBF, cerebral blood flow; IQR, interquartile range; Tm, time-to-maximum of the residue function; HIR, hypoperfusion intensity ratio; CBV, cerebral blood volume; TLKW, time last known well; CTA, CT angiogram; revasc, revascularization; mTICI, modified thrombolysis in cerebral infarction; min, minutes; ICH, intracerebral hemorrhage.

In the univariate analysis, significant differences (p<0.10) between the outcome groups were observed for presenting NIHSS (OR 1.08, 95% confidence interval [CI] 1.01–1.15, p = 0.027), initial glucose (OR 1.02, 95% CI 1.01–1.03, p = 0.001), CTP core size (OR 1.01, 95% CI 1.00–1.02, p = 0.023), CBV index (OR 0.09, 95% CI 0.01–0.99, p = 0.049), treatment with IV thrombolysis (OR 0.38, 95% CI 0.18–0.80, p = 0.012), and post-treatment poor revascularization (mTICI 0-2a) (OR 2.76, 95% CI 0.93–8.20, p = 0.068).

In the multivariable regression analysis, higher presenting NIHSS (OR 1.12, 95% CI 1.02–1.23, p = 0.014), elevated glucose (OR 1.02, 95% CI 1.01–1.03, p = 0.002), absence of treatment with IV thrombolysis (OR 4.01, 95% CI 1.35–11.95, p = 0.013), and poor revascularization (OR 4.76, 95% CI 1.24–18.37, p = 0.023) were independently associated with poor 90-day outcomes (Table 2).

**Table 2:** Multivariable analysis for predicting poor 90-day functional outcomes.

	Odds ratio	OR 2.5% CI	OR 97.5% CI	p-value
NIHSS	1.1219	1.0234	1.2299	0.0142*
Glucose (mg/dL)	1.0207	1.0078	1.0338	0.0016*
CBF < 30%	1.0109	0.9972	1.0247	0.1193
CBV Index	0.0863	0.0036	2.0592	0.1301
Absence of IV Thrombolysis	4.0099	1.3452	11.9534	0.0127*
mTICI 0-2a	4.7641	1.2358	18.3659	0.0234*
TLKW to CTA (min)	0.9998	0.9978	1.0018	0.8168
Age (years)	1.0153	0.9835	1.0482	0.3491

*Significant p-values (p<0.05)

OR, odds ratio; CI, confidence interval; NIHSS, National Institutes of Health Stroke Scale; mg, milligrams; dL, deciliter; CTP, computed tomography perfusion; CBV, cerebral blood volume; IV, intravenous; mTICI, modified thrombolysis in cerebral infarction; TLKW, time last known well; CTA, computed tomography angiography; min, minutes.

Simple logistic regression of the outcome with each prognostic scale indicated that CLEOS (per 25-point increase, OR 1.22, 95% CI 1.10–1.34, p<0.001) and HIAT-2 (per 1-point increase, OR 1.27, 95% CI 1.03–1.55, p = 0.021) were significantly associated with 90-day mRS 4–6, whereas PRE (per 10-point increase, OR 1.17, 95% CI 1.00–1.37, p = 0.052), THRIVE (per 1-point increase, OR 1.21, 95% CI 0.97–1.50, p = 0.089), and SPAN-100 (per 1-point increase, OR 2.13, 95% CI 0.70–6.49, p = 0.184) were not. CLEOS was the only model significantly associated with 90-day mRS 5–6 (per 25-point increase, OR 1.21, 95% CI 1.10–1.33, p<0.001).

**Fig 2:**
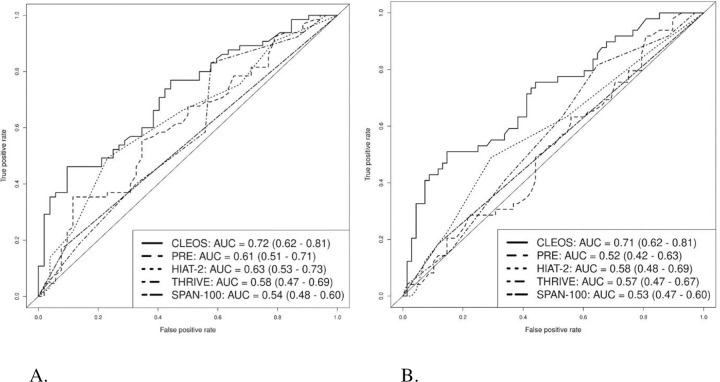
Area under the curve analyses predicting poor functional outcomes. A. ROC curve predicting 90-day mRS 4–6. B. ROC curve predicting 90-day mRS 5–6. ROC, receiver operator characteristics; mRS, modified Rankin Scale; CLEOS, Charlotte Large artery occlusion Endovascular therapy Outcome Score; THRIVE, Totaled Health Risks in Vascular Events; HIAT-2, Houston Intra-Arterial Therapy (HIAT)-2; PRE, Pittsburgh Response to Endovascular therapy; SPAN-100, Stroke Prognostication using Age and NIHSS.

AUC for CLEOS (0.72, 95% CI 0.62–0.81) was statistically superior to PRE (AUC 0.61, 95% CI 0.51–0.71; p = 0.0197), THRIVE (AUC 0.58, 95% CI 0.47–0.69; p<0.001), and SPAN-100 (AUC 0.54, 95% CI 0.48–0.60; p<0.001), and a favorable trend was observed compared to HIAT-2 (AUC 0.63, 95% CI 0.53–0.73; p = 0.0616) for predicting 90-day mRS 4–6 (Fig 2). Similarly, AUC for CLEOS (AUC 0.71, 95% CI 0.62–0.81) was superior to PRE (AUC 0.52, 95% CI 0.42–0.63; p<0.001), HIAT-2 (AUC 0.58, 95% CI 0.48–0.69; p = 0.002), THRIVE (AUC 0.57, 95% CI 0.47–0.67; p<0.001), and SPAN-100 (AUC 0.53, 95% CI 0.47–0.60; p<0.001) for predicting 90-day mRS 5–6 (Fig 2).

**Fig 3:**
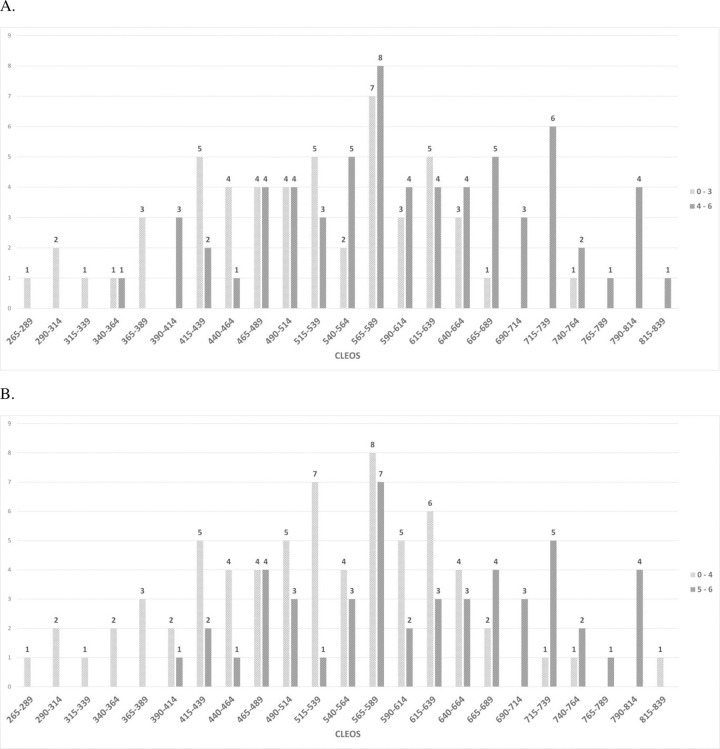
CLEOS scores stratified by functional outcomes. A. CLEOS stratified by mRS 0–3 versus mRS 4–6. B. CLEOS stratified by mRS 0–4 versus mRS 5–6. CLEOS, Charlotte Large artery occlusion Endovascular therapy Outcome Score; mRS, modified Rankin Score.

Nineteen of 20 (95%) patients with CLEOS ≥ 675 had 90-day mRS scores of 4–6, while 10 of 12 (83.3%) with CLEOS ≥ 725 had 90-day mRS scores of 5–6 (Fig 3).

## Discussion

In this retrospective study from the code stroke registry of a large health system, we report that higher initial NIHSS scores, elevated glucose, absence of treatment with IV thrombolysis, and lack of endovascular reperfusion (mTICI 0-2a) are independently associated with poor 90-day functional outcomes in anterior circulation thrombectomy patients presenting with large CTP core infarctions. Also, the previously reported prognostic tools, CLEOS and HIAT-2, predict poor outcomes (90-day mRS 4–6), while CLEOS also predicts devastating outcomes (90-day mRS 5–6), in this population. Given the expanded indications for thrombectomy [8–11], treatment has become the “default” in many scenarios. We thus opted to study poor outcomes, as a high likelihood of a poor outcome may be one factor in the decision to proceed with thrombectomy [14].

Recently published trials have demonstrated the efficacy of thrombectomy for ICA and MCA occlusion patients with large core infarctions [9–11]. While thrombectomy was superior to medical management, median 90-day mRS scores in the treatment groups were 4 in each study. Between 17%-30% of thrombectomy patients were functionally independent at 90 days (mRS 0–2) [9–11]. In contrast, the functional independence rate in a meta-analysis of early window thrombectomy trials was 46% [1]; in late-window thrombectomy trials utilizing patient selection with perfusion imaging mismatch [3] or clinical-core mismatch [2], 90-day functional independence rates were 45% and 49%, respectively.

While it is intuitive that the sub-population of thrombectomy patients presenting with large core infarctions should have lower rates of good outcomes [4], the differences in the proportion of subjects achieving functional independence highlights the importance of pre-thrombectomy risk stratification. For example, a patient meeting entry criterion for SELECT2 would be expected to have an approximately 62% of being non-ambulatory (mRS 4–6) at 90 days with thrombectomy. EVT offers a reasonable opportunity of achieving an mRS score of 0–3 compared with medical management in this scenario (38% vs 19%), recognizing that everyone carries a higher or lower chance of ambulation based on patient-specific factors. While family members are often interested in exhausting any means available for possible improvement of their loved one’s condition [19], risk stratification is also of value before or after emergency medical decision-making as it can set expectations and allow for future planning [14].

Our study is distinguished from SELECT2, ANGEL-ASPECT, and TENSION in that large core infarctions were defined using only CTP criteria. In line with the highest level of guideline recommendations [13], it has not historically been the standard of care to treat patients with ASPECTS < 6 with EVT [13], despite some prior evidence supporting this practice [20, 21]. Accordingly, our findings are not directly comparable with those from recent RCTs. Notable differences in the cohorts include median ASPECTS (4 and 3 in SELECT2 and ANGEL-ASPECT, respectively, compared with 10 in our cohort), median CTP core size (81.5 ml and 60.5 ml in the thrombectomy arms of SELECT2 and ANGEL-ASPECT, respectively, compared with 76.5 ml in our study), and time (> 60% and >70% of patients enrolled outside of the 0-6-hour window in SELECT2 and ANGEL-ASPECT, respectively, compared with 28% in our cohort). Our population is like SELECT2 in that many patients had large volumes of CTP mismatch. In SELECT2, only 50 of 348 patients had mismatch ratios < 1.2 with mismatch volume < 10 ml; median mismatch ratio was 2.15 in our study. Notably, the SELECT2 subgroup with small mismatch still had a greater than 2.5 odds of improved functional outcomes with thrombectomy compared with medical management. Despite these differences, the more favorable subset of large core patients included in our study (preserved ASPECTS, large CTP core) offers insight into prognosticating in a “less favorable” subset; a patient predicted to have a poor outcome based on our findings has a high likelihood of a poor outcome in a broader large core patient population. This is supported by the higher 90-day mRS 4–6 rate reported in SELECT2 (61.8%) versus our study (55.9%).

In our analysis, only 1 of 20 large core patients by CTP criteria with CLEOS ≥ 675 was ambulatory at 90 days (Fig 2), and 10 of 12 with CLEOS ≥ 725 had a 90-day mRS score of 5–6. While we do not advocate making medical decisions based solely on any scale, the combination of data from phase III clinical trials [8–11], patient-specific factors [14], and overall goals of care may play a part in patient risk stratification. A high CLEOS score may provide some additional prognostic information for families as they weigh pre- and post-thrombectomy medical decision making, providing a conservative estimate of expected poor outcomes, given the more favorable characteristics of our large core cohort. The findings of this study are also in line with a previous analysis that included a broad population of ICA and MCA thrombectomy patients [14]. In that study, patients with CLEOS ≥ 700 did not derive a statistically significant benefit from excellent revascularization to avoid a 90-day mRS score of 4–6 [14]. Based on these analyses, a high CLEOS score, in the range of 675 or above, should prompt discussion with a patient’s family about the potential expected outcome. The distribution of functional outcomes, stratified by CLEOS score, is further illustrated in Fig 3.

Presenting NIHSS [14–18], initial glucose [14, 15], and revascularization [22, 23] are well-described stroke prognostic factors, and were also independent predictors of outcome in our study. Intravenous thrombolysis, an evidence-based treatment for acute stroke patients presenting within 4.5 hours or for those with mismatch on acute stroke imaging [13], was also an independent predictor of outcome. A potential rationale for the benefit of IV thrombolysis is the large proportion of subjects with ischemic penumbra in our cohort, which had a median mismatch volume of 91.5 ml. Patients with evidence of penumbra benefit from treatment with IV alteplase, as shown in prior randomized trials [24, 25]. Moreover, late-window IV thrombolysis with Tenecteplase in patients with ischemic penumbra has proven to be safe but not efficacious [26].

Our study has several limitations, namely the retrospective design that subjects the findings to bias. As mentioned, our data only include a subset of the population studied in SELECT2 and ANGEL-ASPECT and are not representative of all enrolled patients. However, this might offer insight into the chances of a poor outcome in large core patients with more favorable imaging profiles. The more stringent pre-morbid functional status inclusion criteria for SELECT2 and ANGEL-ASPECT (mRS 0–1), compared to the general consideration of patients with pre-morbid mRS 0–2 for thrombectomy in our health system and in other trials [1, 11], likely impacts rates of poor 90-day outcomes in each of the respective populations. CTP was performed with either RAPID or Viz.ai, so variability in core sizes was possible based on the processing software used. Core size may be overestimated on CTP by CBF < 30% volume for ultra-early presenting patients [27], though this parameter was chosen as it is the standard definition for core size and has been used in multiple randomized trials [2, 3, 9, 10]. Thrombectomy eligibility was based on our health system criteria, which may differ among institutions, though are based on recommendations from the American Heart Association/American Stroke Association. Thus, our results do not directly apply to alternate potential thrombectomy patients, such as those with low presenting NIHSS scores or more distal vessel occlusions. Relatively small numbers of patients had very high CLEOS scores, so firm conclusions regarding these subjects require more investigation. Not all studied prognostic scores were designed to predict mRS 4–6 and mRS 5–6, though these were chosen as standard poor outcomes for this analysis. Included subjects were all treated with thrombectomy, so a medical comparison group was not evaluated, subjecting the study to selection bias. Lastly, our findings would be strengthened by replication in alternate and larger data sets.

## Conclusion

We report that thrombectomy patients with large CTP core infarctions and higher presenting stroke severity, elevated glucose, absence of treatment with IV thrombolysis, and those not achieving endovascular reperfusion have a higher likelihood of poor functional outcomes. The previously reported prognostic tool CLEOS can identify patients at high likelihood of poor or devastating outcomes in a favorable subset of the population enrolled in recently published large core randomized trials. Future studies may explore predictive models in other large core patient cohorts and investigate the role of prognostic tools in acute stroke decision-making.

## Supporting information

S1 Table Health system endovascular thrombectomy guideline. NIHSS, National Institutes of Health Stroke Scale; mRS, modified Rankin Scale; ICA, internal carotid artery; M1, middle cerebral artery first segment; M2, middle cerebral artery second segment; ASPECTS, Alberta Stroke Program Early Computed Tomography Score.
(DOCX)

S2 Table Stroke outcome prediction scales. NIHSS, National Institutes of Health Stroke Scale; mg, milligrams; dL, deciliters; CBV, cerebral blood volume; mRS, modified Rankin Scale; mTICI, modified thrombolysis in cerebral infarction; ASPECTS, Alberta Stroke Program Early Computed Tomography Score; EVT, endovascular thrombectomy.
(DOCX)
