# Correction: Predicting poor functional outcomes for patients with large computed tomography perfusion core infarctions treated with endovascular thrombectomy

**DOI:** 10.1371/journal.pone.0351479

**Published:** 2026-06-12

**Authors:** The PLOS One Editors

## Notice of Republication

This article [1] was republished on April 24, 2026, to address an issue identified post-publication. An updated version of S1 Raw data is provided with this notice. Please download this article again to view the correct version.

The article’s Data Availability statement is updated to: All relevant data are within the paper and its Supporting Information files.

With this Correction the *PLOS One* Editors inform readers that there was a potential competing interest between the authors and one or more people involved in peer review. After reviewing this matter PLOS concluded that the article’s publication is supported based on expert input that was not affected by the concern. We regret that the issues were not addressed prior to the article’s publication.

## Supporting information

S1 Raw data (XLSX)
