# Supplementary material for: Predicting poor functional outcomes for patients with large computed tomography perfusion core infarctions treated with endovascular thrombectomy
Source: PLoS One. 2024 Nov 18;19(11):e0309163. doi: 10.1371/journal.pone.0309163 (PMC11573161; doi:10.1371/journal.pone.0309163)
Supplement: S1 Table — NIHSS, National Institutes of Health Stroke Scale; mRS, modified Rankin Scale; ICA, internal carotid artery; M1, middle cerebral artery first segment; M2, middle cerebral artery second segment; ASPECTS, Alberta Stroke Program Early Computed Tomography Score. (DOCX) [file pone.0309163.s001.docx]

**Supplemental Table 1. Health System Endovascular Thrombectomy Guideline**

| 0 – 6 Hours | 6 – 24 Hours |
| --- | --- |
| NIHSS ≥ 6 or disabling deficit | NIHSS ≥ 6 or disabling deficit |
| mRS 0 - 2 | mRS 0 - 2 |
| ICA, M1, proximal M2 occlusion | ICA, M1, proximal M2 occlusion |
| ASPECTS ≥ 6 | ASPECTS ≥ 6 |
|  | Core infarct < 70 cc  Mismatch volume ≥ 15 cc  Mismatch ratio ≥ 1.8 |

NIHSS, National Institutes of Health Stroke Scale; mRS, modified Rankin Scale; ICA, internal carotid artery; M1, middle cerebral artery first segment; M2, middle cerebral artery second segment; ASPECTS, Alberta Stroke Program Early Computed Tomography Score.
