# Supplementary material for: Predicting poor functional outcomes for patients with large computed tomography perfusion core infarctions treated with endovascular thrombectomy
Source: PLoS One. 2024 Nov 18;19(11):e0309163. doi: 10.1371/journal.pone.0309163 (PMC11573161; doi:10.1371/journal.pone.0309163)
Supplement: S2 Table — NIHSS, National Institutes of Health Stroke Scale; mg, milligrams; dL, deciliters; CBV, cerebral blood volume; mRS, modified Rankin Scale; mTICI, modified thrombolysis in cerebral infarction; ASPECTS, Alberta Stroke Program Early Computed Tomography Score; EVT, endovascular thrombectomy. (DOCX) [file pone.0309163.s002.docx]

**Supplemental Table 2. Stroke Outcome Prediction Scales^#^**

| Scale Name | Calculation | Interpretation |
| --- | --- | --- |
| Charlotte Large artery occlusion Endovascular therapy Outcome Score (CLEOS)^1^ | (5 x age [years]) + (10 x NIHSS) + Glucose (mg/dL) – (150 x CBV index) | Higher scores associated with poor outcome (90-day mRS 4-6). Scores ≥ 700 associated with no statistical benefit of excellent endovascular reperfusion (mTICI 2c-3) in original derivation manuscript. |
| Totaled Health Risks in Vascular Events (THRIVE)^2^ | Age 60-79 years, 1 point; age ≥80 years, 2 points; NIHSS 11-20, 2 points; NIHSS ≥21, 4 points; hypertension, diabetes mellitus, atrial fibrillation, 1 point each | Points (range 0-9)  Score 6-9 associated with poor 90-day outcomes. |
| Houston Intra-Arterial Therapy (HIAT)-2^3^ | Age ≤59 years, 0 points; age 60-79 years, 2 points; age ≥80 years, 4 points; glucose < 150 mg/dL, 0 points, glucose ≥150 mg/dL, 1 point; NIHSS ≤10, 0 points; NIHSS 11-20, 1 point; NIHSS ≥21, 2 points; ASPECTS 8-10, 0 points; ASPECTS ≤7, 3 points | Points (range 0-10)  Score ≥5 associated with poor 90-day outcomes. |
| Pittsburgh Response to Endovascular therapy (PRE)^4^ | Age (years) + (2 x NIHSS) – (10 x ASPECTS) | -25 to +49, likely to benefit from successful EVT; ≥ 50 not likely to benefit from successful EVT. |
| Stroke Prognostication using Age and NIHSS (SPAN-100)^5^ | Age (years) + NIHSS | Score ≥100 associated with poor outcomes. |

#Reproduced with permission from World Neurosurgery (Karamchandani RR, Satyanarayana S, Yang H, et al. The Charlotte Large Artery Occlusion Endovascular Therapy Outcome Score Predicts Poor Outcomes 1 Year After Endovascular Thrombectomy. *World Neurosurg* 2023; 173: e415-e421.

NIHSS, National Institutes of Health Stroke Scale; mg, milligrams; dL, deciliters; CBV, cerebral blood volume; mRS, modified Rankin Scale; mTICI, modified thrombolysis in cerebral infarction; ASPECTS, Alberta Stroke Program Early Computed Tomography Score; EVT, endovascular thrombectomy

^1^ [Karamchandani RR, Prasad T, Strong D, et al. A tool to improve stroke outcome prediction: The charlotte large artery occlusion endovascular therapy outcome score. *J Stroke Cerebrovasc Dis* 2022; 31: 106393.](http://paperpile.com/b/Q8VdUd/YTDm)

^2^ [Flint AC, Cullen SP, Faigeles BS, et al. Predicting Long-Term Outcome after Endovascular Stroke Treatment: The Totaled Health Risks in Vascular Events Score. *American Journal of Neuroradiology* 2010; 31: 1192–1196.](http://paperpile.com/b/Q8VdUd/hGZF)

^3^ [Sarraj A, Albright K, Barreto AD, et al. Optimizing prediction scores for poor outcome after intra-arterial therapy in anterior circulation acute ischemic stroke. *Stroke* 2013; 44: 3324–3330.](http://paperpile.com/b/Q8VdUd/FnHf)

^4^ [Rangaraju S, Aghaebrahim A, Streib C, et al. Pittsburgh Response to Endovascular therapy (PRE) score: optimizing patient selection for endovascular therapy for large vessel occlusion strokes. *J Neurointerv Surg* 2015; 7: 783–788.](http://paperpile.com/b/Q8VdUd/uaBT)

^5^ [Saposnik G, Guzik AK, Reeves M, et al. Stroke Prognostication using Age and NIH Stroke Scale: SPAN-100. *Neurology* 2013; 80: 21–28.](http://paperpile.com/b/Q8VdUd/cl8S)
